# Necrotizing fasciitis and meningitis due to *Streptococcus pneumoniae* serotype 9 N: a case report

**DOI:** 10.1186/s12879-019-3969-4

**Published:** 2019-04-29

**Authors:** Nichlas Hovmand, Sarah Byberg, Morten Bo Larsen, Daria Podlekareva, David Levarett Buck, Birgitte Rønde Hansen

**Affiliations:** 1 Department of Infectious Diseases, Copenhagen University Hospital, Hvidovre, Denmark; 2 Department of Endocrinology, Copenhagen University Hospital, Hvidovre, Denmark; 3 Department of Orthopedics, Copenhagen University Hospital, Hvidovre, Denmark; 4 Department of Infectious Diseases, Copenhagen University Hospital, Rigshospitalet, Denmark; 5 Department of Intensive Care, Copenhagen University Hospital, Rigshospitalet, Denmark

**Keywords:** Necrotizing fasciitis, Meningitis, *Streptococcus pneumoniae*, Infection

## Abstract

**Background:**

Necrotizing fasciitis is a deep infection of the fascia and subcutaneous tissue with a high mortality rate. Meningitis is an infection of the membranes surrounding the brain with a likewise high mortality rate. *Streptococcus pneumoniae* is the most frequent cause of bacterial meningitis and it is an extremely rare cause of necrotizing fasciitis. Different subcapsular serotypes of *S. pneumoniae* are known to have diverse virulence. The serotype 9 N is associated with a high risk of death.

**Case presentation:**

We report a case of a previously healthy 68-year-old female who presented at our clinic with complaints of pain in her left calf since having experienced a very painful leg cramp 3 weeks prior. Within a few hours after admission, she developed fever, neck stiffness and an altered mental state. Concurrently, the pain in her leg worsened. Upon further examination it was found that she suffered from both meningitis and necrotizing fasciitis due to *S. pneumoniae*, serotype 9 N. The patient survived and avoided leg amputation.

**Conclusions:**

The patient suffered from two very lethal infections simultaneously. Both of them were caused by *S. pneumoniae*. We believe that her favorable outcome was, a result of prompt surgical intervention and appropriate antibiotic treatment. Our case underlines the importance of continuous reevaluation of the symptoms and clinical findings in patients with unclear causes of severe illness, especially if the patient’s condition changes.

## Background

Necrotizing fasciitis (NF) is an infection which may involve tissue extending from the epidermis to the deep musculature and result in widespread tissue destruction. It was first described in 1952 [1] and is characterized by rapid progression, severe pain and high mortality [2]. During surgery, findings of a friable fascia and dish-water grey exudate make the diagnosis. The treatment consists of appropriate antibiotic treatment and surgical débridement where time to treatment initiation is of crucial importance. The Laboratory Risk Indicator for Necrotizing Fasciitis (LRINEC) score that is based on routine laboratory tests (Table 1), can aid clinicians in recognizing patients with soft tissue infections who have a high risk of developing NF [3]. However, the score’s positive- and negative predictive value for differentiating NF from cellulitis in the emergency department are both poor [4].

**Table 1:** LRINEC score

Test		Points
C-reactive protein (mg/L)	< 150	0
	> 150	4
White blood cell count (×10^9^/L)	< 15	0
	15–25	1
	> 25	2
Hemoglobin (g/dL)	> 13.5	0
	11–13.5	1
	< 11	2
Sodium (mmol/L)	> 135	0
	< 135	2
Creatinine (μmol/L)	< 141	0
	> 141	2
Glucose (mmol/L)	< 10	0
	> 10	1

Patients with soft tissue infection and an LRINEC score of 6 or higher should be carefully evaluated for the presence of necrotizing fasciitis

Bacterial meningitis is another highly lethal infection classically characterized by a triad of symptoms: Fever, neck stiffness and altered mental status. Other symptoms associated with meningitis are photophobia, nausea and headache. However, the diagnosis can be difficult to make in the initial phase as the initial symptoms usually are very unspecific. Patient history and clinical examination can neither confirm nor exclude meningitis [5]. A spinal tap is needed to ensure the accurate diagnosis. Diagnostic tests and appropriate antibiotic treatment must be initiated quickly, as the infection can progress from mild symptoms to a life-threatening condition in a matter of hours.

*Streptococcus pneumoniae* (*S. pneumoniae*) is a common cause of pneumonia and meningitis [6]. It is an extremely rare cause of NF. We conducted a search in PubMed of the literature describing NF caused by *S. pneumoniae* which yielded 14 case reports published between 2001 and 2016 including a total of 17 patients [7–17]. At least 3 of the 17 patients had suffered a blunt trauma to the tissue in which NF later developed [18–20]. In 4 of the 17 cases the serotype of the bacteria was mentioned. There were reports of 2 cases of serotype 9 V [14, 18], 1 case of serogroup 9 [20] and 1 case of serogroup 5 [17]. None of the cases specifically reports serotype 9 N. The mortality rate from invasive *S. pneumoniae* infection depends on the specific *S. pneumoniae* subcapsular serotype. Serotype 9 N is associated with a higher than average mortality from invasive *S. pneumoniae* infection [21, 22].

## Case presentation

A 68-year-old Caucasian female was admitted to our clinic, with suspected deep venous thrombosis, presenting with pain in her left calf that had lasted for 3 weeks starting after a very painful nocturnal leg cramp, and worsened over the last few days. At the time of admission, she had also experienced 5 days of malaise and loss of appetite and in addition 1 day of headache and lower back pain. The patient had been coughing a lot ever since recovering from a confirmed Influenza B upper airway infection 3 months earlier. The patient received antihypertensive medication but had no other known chronical medical conditions.

**Fig. 1:**
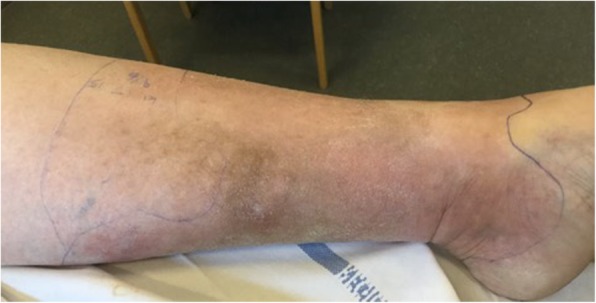
The patient’s left leg before the operation

At the time of admission, she was fully awake with a Glasgow Coma Scale (GCS) of 15. She was afebrile with a temperature of 36.3 °C (97.3 F) and had no neck stiffness. She presented with a well-defined erythema on her left calf (Fig. 1) and any touch or movement of her left knee, ankle or calf was very painful. There were no clinical signs of intra-articular fluid in the knee.

Her blood tests showed a very high level of C-reactive protein (CRP) of 450 mg/L (normal range below 10 mg/L) and D-dimer of 18 FEU/L (normal range below 0.5 FEU/L). Her LRINEC score was 5 points (4 points from CRP 450 mg/L and 1 point from hemoglobin 12.9 g/dL (normal range between 12.1 g/dL – 15.1 g/dL)). Treatment with cefuroxime was initiated due to suspected infection of unknown origin. A CT scan of the abdomen and thorax performed due to suspected lung embolism showed discrete bilateral atelectatic areas of the lungs, but otherwise normal findings.

Four hours after admission the patient had developed an altered mental state with GCS of 9 (Eyes 2, Verbal 2 and Motor 5), neck stiffness and a fever with a temperature of 38.8 °C (102 F). Due to suspected meningitis a spinal tap was performed and empiric treatment against meningitis was initiated with Dexamethasone, Ceftriaxone and Ampicillin. The tests of the cerebrospinal fluid (CSF) showed signs of meningitis with a white blood cell count (WBC) of 50 cells/μL that were 81% granulocytes, very high lactate of 17 mmol/L, very low glucose of 0.3 mmol/L and very high protein level above 6 g/L. Initial microscopy showed Gram-positive diplococci. A CT-scan of the patient’s brain was performed and showed no abnormalities.

**Fig. 2:**
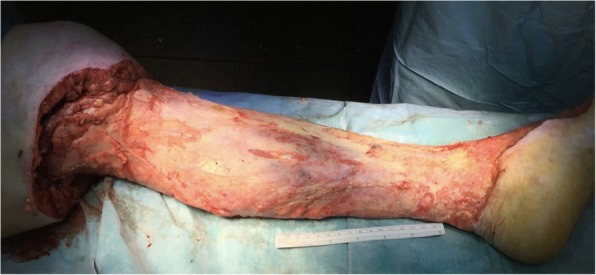
The patient’s left leg after the operation

Eight hours after admission the patient showed a slight decline in GCS of 8 (Eyes 2, Verbal 2 and Motor 4). However, she still showed a disproportionally strong pain reaction to any touch of the left calf. The erythema had not spread since admission to the hospital, but over the next 2 h a spread of about 5 cm proximally was observed. Due to the progression of the erythema together with the strong pain reaction despite a decline in mental state, a diagnostic incision of the tissue was performed in local analgesia. The subcutis was found to be loose from the fascia and the tissues looked nonvital. Antibiotic treatment was changed to Meropenem, Clindamycin and Ciprofloxacin against NF due to national guidelines. An acute surgical débridement was performed (Fig. 2). The patient was transferred to a hospital specialized in the treatment of patients with NF, including specialized surgical care, intensive care, and hyperbaric oxygen treatment. Reinspection of the tissue in the left leg was performed with no findings of necrotic tissue. Inspection of the leg was afterwards performed routinely once daily with normal findings.

Over the next days, *S. pneumoniae* was cultured both in the CSF and in 4 out of 4 blood cultures from the patient. The serotype of the bacteria was determined as 9 N. No bacteria were found by culturing the tissue from the leg, but 16S-PCR of the tissue was performed, which also showed *S. pneumoniae*. The 16S-PCR was performed at a department of clinical microbiology at a big university hospital. It was performed from a commercially available MicroSEQ kit with a fragment size of 500 base pairs. The cultured *S. pneumoniae* were susceptible to all antibiotics that were tested, including benzylpenicillin. No other bacteria were found. Therefore, antibiotic treatment was switched to benzylpenicillin on day 3 after admission.

**Fig. 3:**
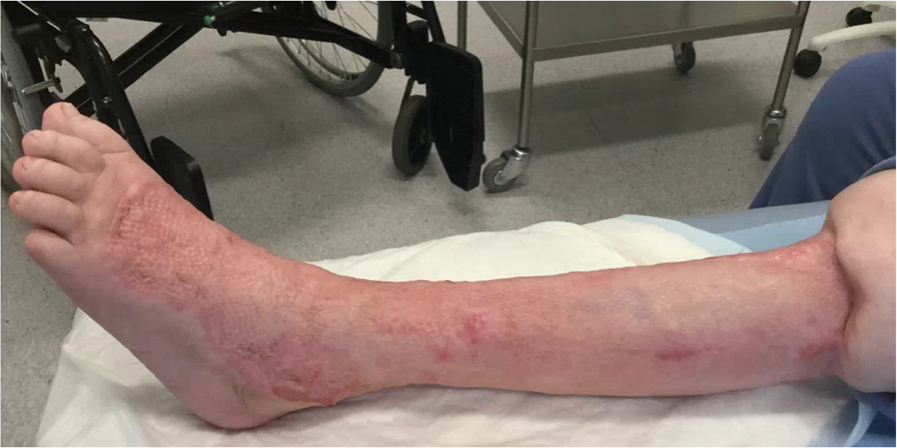
The patient’s left leg after reconstructive surgery

The patient subsequently underwent reconstructive surgery with a split skin graft (Fig. 3) and was discharged after 51 days of hospitalization, including in departments of intensive care, infectious diseases and plastic surgery.

The patient later underwent different tests for immunodeficiency. Immunoglobulins, HIV-test and differential WBC count were all normal. The patient was not vaccinated against *S. pneumoniae* prior to her admission. However, she has been afterwards and serotype 9 N is included in the vaccine.

## Discussion

We here present a case of a previously healthy, unvaccinated, adult female who suffered from disseminated *S. pneumoniae* infection causing both meningitis and NF. The initial infection with *S. pneumoniae* is believed to stem from the respiratory tract, as she had been coughing for several months prior to admission. Furthermore, the CT scan performed upon admission showed atelectatic areas of the lungs. It is possible that the lungs were rendered vulnerable after an influenza infection, in turn permitting a superinfection with *S. pneumoniae*. The *S. pneumoniae* serotype, 9 N is known to be very virulent and must have spread to the bloodstream and further on to the CSF causing meningitis [21, 22]. Remarkably, in this case the bacteria also caused NF of the leg, which developed over a very similar time course to the meningitis. Curiously, at least 3 of the earlier reported cases of *S. pneumoniae* NF describe a blunt trauma prior to infection [18–20]. Blunt traumas has previously been found to be a risk factor for developing NF [23]. The patient in our case experienced a very painful nocturnal leg cramp 3 weeks prior to the infection. We hypothesize that the cramp could mimic a blunt trauma creating a favorable local environment that enabled development of *S. pneumoniae* NF. Other than medically treated hypertension the patient had no known medical conditions and no immunodeficiencies could be demonstrated on later examination. We believe that the virulence of the 9 N serotype of *S. pneumoniae* combined with a trauma equivalent were determining for the chain of events in this case. Given the rarity of *S. pneumoniae* NF and how even more unlikely concurrent meningitis is, we were initially uncertain whether they were in fact the correct diagnoses. However, due to the classical presentation of both conditions, ie. operative findings and severe pain for NF and the triad of symptoms: Fever, neck stiffness and altered mental status, for meningitis taken together with the rapidness of symptom progression, we concluded that the patient did indeed suffer from both conditions. The LRINEC score of 5 points underlines that a scoring system like LRINEC can be used to support or raise a suspicion of NF, albeit not stand alone to determine whether or not a patient has NF.

## Conclusions

The patient in our case suffered from two concurrent and very lethal infections caused by *S. pneumoniae*. Fortunately, the patient both survived and avoided leg amputation due to sufficient surgical debridement and relevant antibiotic treatment initiated in an early stage as well as appropriate changes made to the treatment as new information emerged. The mortality rate of patients with NF is extremely high and early diagnosis, prompt surgical intervention and appropriate antibiotic treatment are essential to reduce mortality and improve outcome.

Making the correct diagnosis even with classical symptoms of known medical conditions can be difficult in the initial phases of the disease. Our case underlines the importance of continuously reevaluating the symptoms and clinical findings in patients with unclear causes of severe illness, especially if the patient’s condition changes.

## Abbreviations

CRP: C-reactive protein
CSF: Cerebrospinal fluid
GCS: Glasgow Coma Scale
LRINEC: Laboratory risk indicator for necrotizing fasciitis
NF: Necrotizing Fasciitis
*S*.: *Streptococcus*
WBC: White Blood Cell
